# GNSS-Assisted Integrated Sensor Orientation with Sensor Pre-Calibration for Accurate Corridor Mapping †

**DOI:** 10.3390/s18092783

**Published:** 2018-08-24

**Authors:** Yilin Zhou, Ewelina Rupnik, Paul-Henri Faure, Marc Pierrot-Deseilligny

**Affiliations:** 1 LaSTIG, IGN, ENSG, University Paris-Est, F-94160 Saint-Mande, France; Ewelina.Rupnik@ign.fr (E.R.); Marc.Pierrot-Deseilligny@ensg.eu (M.P.-D.); 2 CACOH, Compagnie Nationale du Rhône, 69007 Lyon, France; P.FAURE@cnr.tm.fr

**Keywords:** photogrammetry, sensor calibration, GNSS, integrated sensor orientation

## Abstract

With the development of unmanned aerial vehicles (UAVs) and global navigation satellite system (GNSS), the accurate camera positions at exposure can be known and the GNSS-assisted bundle block adjustment (BBA) approach is possible for integrated sensor orientation (ISO). This study employed ISO approach for camera pose determination with the objective of investigating the impact of a good sensor pre-calibration on a poor acquisition geometry. Within the presented works, several flights were conducted on a dike by a small UAV embedded with a metric camera and a GNSS receiver. The multi-lever-arm estimation within the BBA procedure makes it possible to merge image blocks of different configurations such as nadir and oblique images without physical constraints on camera and GNSS antenna positions. The merged image block achieves a better accuracy and the sensor self-calibrated well. The issued sensor calibration is then applied to a less preferable acquisition configuration and the accuracy is significantly improved. For a corridor acquisition scene of about 600 m, a centimetric accuracy is reached with one GCP. With the provided sensor pre-calibration, an accuracy of 3.9 cm is achieved without any GCP.

## 1. Introduction

For traditional airborne photogrammetry with unmanned aerial vehicles (UAVs), camera poses (position and attitude) are determined indirectly using the well-known method bundle block adjustment (BBA). The BBA method is effective and widely employed for photogrammetric production when the scene is well-textured and allows for automated tie point extraction.

The photogrmmetric accuracy is strongly dependent on the acquisition geometry and the number of ground control points (GCPs) as well as their distribution within the image block [1,2]. Among different configurations of UAV acquisitions, the corridor mapping is of particular interest, for instance in dike surveillance, highway planning and power line surveys [3]. Nevertheless, it is challenging in many aspects. The challenges come mainly from the special network geometry, and the fact that a well-distributed GCP network is not easy to ensure. The acquired images are often in strips, which makes it difficult to have satisfying cross-track overlaps and thus results in a less accurate camera pose determination. While the employment of a large number of GCPs prevents stereo model distortion [4], the field work of GCP establishment can be substantially expensive and time-consuming.

With the appearance and development of global navigation satellite system (GNSS), it is possible to know the accurate camera projection center positions at exposure and thus to reduce, or even eliminate, the requirement of GCPs within the image block [5,6]. Several GCPs are still used for the purpose of improving the redundancy and identifying possible bias in GNSS positioning [7,8]. When coupling this GNSS-assisted bundle block adjustment approach to an inertial navigation system (INS), camera poses can be determined directly for each image without the need of traditional BBA procedure. This said, for small UAVs, an accurate position and attitude aerial control remains challenging due to the limited availability of payload, power and volume.

### 1.1. Related Work

Different studies have been carried out to mitigate the errors in INS/GNSS system. The linear offset lever-arm can be determined indirectly by computing the difference between GNSS-derived positions of the antenna reference point and the camera projection centers issued from bundle block adjustment [9]. This offset can also be estimated as an additional parameter during the bundle block adjustment, however, the accuracy is limited due to its correlation with camera interior orientation parameters [10,11,12]. While, with classical methods (by a calliper or by photogrammetric approaches), the linear offset lever-arm can be determined, it is not possible for the boresight calibration to reach sufficient accuracy in the same way. Accordingly, several boresight calibration methods have been proposed [13,14,15,16]. It can be performed either with “one-step” method (i.e., estimation within the BBA process) or with “two-step” method (i.e., comparison of the INS/GNSS-derived attitude with that obtained by BBA process). Moreover, taking into account the remaining temporal correlations within the navigation system can lead to a more realistic estimation [17]. Despite the possibilities of improving the INS/GNSS system accuracy, the INS system demands more effort to reduce errors and often needs accurate GNSS positions as constraints for error control. Given the limited UAV payload capability, this demand on accuracy and thus on high quality of INS/GNSS sensors can be difficult to meet.

Therefore, for small UAV photogrammetric acquisitions, the position aerial control of GNSS systems outperforms the attitude aerial control of INS systems with its better accuracy, lower cost and lower payload requirements. The assisted bundle block adjustment with GNSS data, tie points and GCPs seems to be a more interesting approach. This approach is also referred to as Integrated Sensor Orientation (ISO). On the other hand, due to the substantial inconvenience and cost of GCP establishment, especially in areas with difficult access, the number of GCPs is to be reduced to a minimum.

In 2002, a multi-site test investigating sensor orientation is carried out by the European Organization for Experimental Photogrammetric Research (OEEPE) [18] using the state-of-the-art GNSS/IMU technology of 1999. It shows that the direct sensor orientation can reach an accuracy of 5–10 cm in planimetry and 10–15 cm in altimetry, which is larger than the standard photogrammetric result by a factor of 2–3. The additional introduction of tie points into BBA procedure without GCPs improves in particular the accuracy in height and an accuracy of 5–10 cm is reached. If a minimum of GCPs is introduced, one can achieve an accuracy in object space very similar to that of conventional photogrammetry. Though with direct sensor orientation, the time and cost decrease significantly, integrated sensor orientation is preferable when very high accuracy is indispensable.

In [19], an aerial acquisition of block configuration is performed on a 250 m×300m block with an embarked GNSS-inertial system integrated with a Sony a7R camera. Eight north–south strips and one cross strip are flown at 80m, resulting in a GSD of 1 cm. With aerial control issued from the GNSS-inertial system and one GCP included in the BBA procedure, an accuracy of 3 cm is obtained in both horizontal axis and 11 cm in vertical component.

For a corridor mapping, two flight strips are flown at 135 m [20]. Data of a high-grade dual-frequency GPS receiver are introduced in BBA procedure as aerial position control. With pre-calibrated camera and without GCPs, an accuracy of 12, 11 and 64 cm are reached in northing, easting and height components, respectively.

With the aid of precise aerial position control together with a pre-calibrated camera, two case studies for accurate mapping are presented without GCPs [5]. The first one being block configuration, a self-calibration is performed within the BBA procedure without GCPs. The obtained accuracy with this configuration is 4.3 cm horizontal and 4 cm vertical. The second case study is a corridor configuration of 1200×180m and the camera is pre-calibrated. Without GCPs in BBA procedure, an accuracy of 5.9, 3.3 and 7.0 cm is reached on *x*, *y* and *z* components, respectively.

A new approach concerning relative aerial control is proposed for integrated sensor orientation in [21]. To some extent, the procedure is simpler since the boresight matrix vanishes from the model; moreover, high-grade dual-frequency GNSS receiver can be replaced by single-frequency and carrier-phase GNSS receiver since relative aerial control is more resistant to GNSS bias. A block configuration is carried out with relative aerial control [6]. With one GCP and partially biased GNSS data, the accuracy is maintained compared to without GNSS bias and is of 2.9, 2.2 and 3.8 cm on *x*, *y* and *z* components, respectively.

An dynamic network is proposed to tightly integrate GNSS/INS measurements into BBA procedure in [22]. For a two-flight-strip acquisition of corridor configuration with pre-calibrated boresight, lever-arm and camera intrinsics, an accuracy of 2.5, 1.5 and 1.2 cm on easting, northing and vertical components, respectively, is obtained without GCPs.

### 1.2. Paper Structure

Section 2 gives brief introduction of the employed UAV system and sensors. Section 3 gives details on the methodological aspect. In Section 4, the nature of the acquisition field is described and the conducted flights are presented. Section 5 is devoted to details on the data processing. Finally, the results of conducted acquisitions are presented and the photogrammetric accuracy is evaluated. Discussion of the results are given in Section 6, as well as recommendations for further investigations.

## 2. System Design

### 2.1. UAV

**Figure 1:**
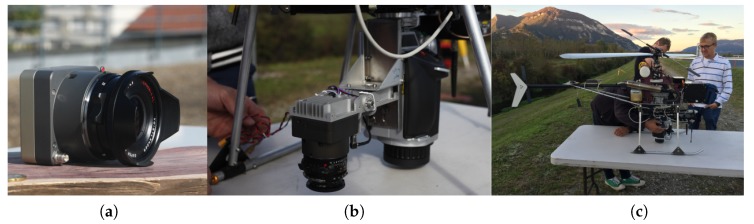
CamLight (**a**); camera set-up on UAV (**b**); and UAV (**c**).

The chosen UAV is a Copter 1B of SURVEY Copter (see Figure 1). It has a wingspan of 1.82 m and a length of 1.66 m. Powered by a gasoline engine, the maximal payload capacity of the UAV is 4.1 kg and the endurance is up to 60 min. The nominal flying altitude of the UAV is 150 m and the maximal flight speed is 10 ms. The UAV possesses a radio communication with its command station. Given a pre-set flight plan registered in the command station, the flight can be performed automatically. Thus, a steady longitudinal/lateral overlap can be assured. An aluminium base mounted on the UAV was adopted for rigid camera installation and cable fixation.

### 2.2. Camera

The camera chosen for data acquisition is an in-house metric camera CamLight (see Figure 1), designed by team Loemi (Laboratoire d’Opto-éléctronique, de Métrologie et d’Instrumentation) of laboratory LaSTIG, IGN (Institut National de l’Information Géographique et Forestière) to meet the needs of photogrammetric UAV acquisitions [23]. The compact camera body (without lens) has a low mass of 160 g and is compatible with most commercially available lenses. The camera is equipped with a full frame sensor of 5120×3840 pixels and a 35 mm lens (140 g). During acquisition, the camera is powered by the on-board power supply and is triggered with an intervalometer every 2.5 s.

### 2.3. GNSS Module

The GNSS module chosen to be integrated to the camera system is a u-blox Neo M8T chip with a L1 GPS antenna. The GPS time of exposures is provided by the GPS module and is registered in the header file for each image. During the acquisition, the GPS sampling rate was set to 1Hz.

## 3. Methodology

**Figure 2:**
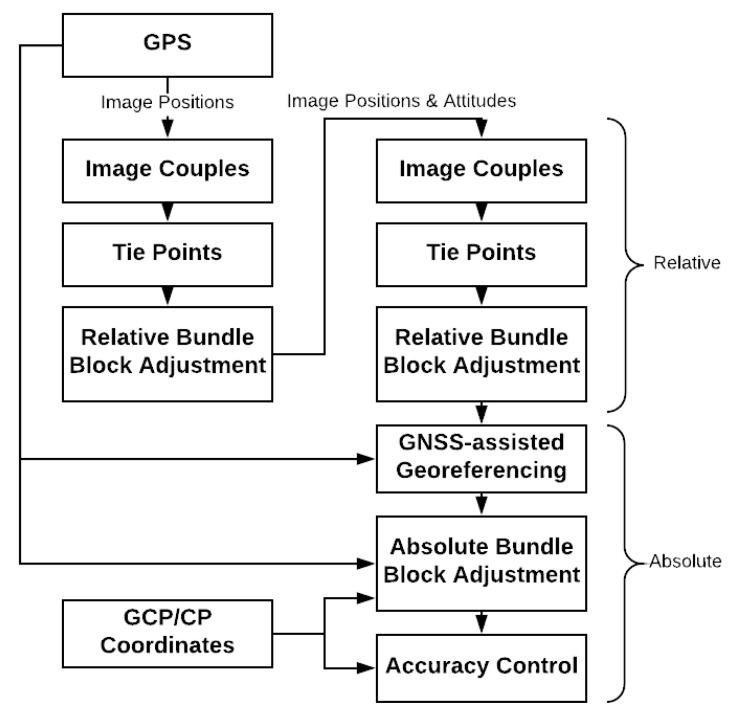
Data processing workflow.

Figure 2 depicts the global data processing workflow. With the aid of a priori information coming from GNSS trajectories, tie points were extracted solely on overlapping images with the algorithm SIFT (Scale Invariant Feature Transform) [24]. A first BBA procedure including only tie points was performed to recover the observed scene. At this step, we get information of the camera attitudes and the tie points are completed by taking this into account. A second BBA procedure including complete tie points was performed for estimating camera poses in a relative scale. GNSS-derived camera positions were used to transform the estimated relative camera poses to absolute ones by estimating a spatial similarity. Afterwards, the tie points, the GCPs, the GNSS-derived camera positions and the image measurements were used as observations for an absolute BBA. Finally, the accuracy was evaluated with CPs.

The bundle block adjustment was carried out with bootstrap solutions. Performing direct algorithm on a single image, a pair or a triplet of images, the global orientation was deduced sequentially starting from a seed image pair [25]. The camera was self-calibrated during the relative BBA process with a two-step procedure. Firstly, the center of distortion (CD) and the radial symmetric distortion (RSD) were estimated (the latter with a polynomial up to $R^{15}$). Then, the RSD and the CD parameters were fixed, and the asymmetric radial distortion was estimated. The objective of the second phase was to model the sensor and lens discrepancies that have no physical explanations. The refined camera model, especially the high degree polynomial for radial distortion correction, mitigates significantly the recurrent “dome effect” without over-parameterizing [4,26]. The estimated parameters of the camera model then served as input for an absolute bundle block adjustment and can be either re-estimated or fixed within the procedure. This absolute BBA procedure takes tie points, GNSS-derived camera positions, GCP coordinates and image measurements as observations. Aforementioned observations being redundant, the cost function was minimized with least square method.

The mathematical model of the performed bundle block adjustment is presented as follows. The cost function to minimize is:

(1) $$\begin{array}{cc} E & =\sum_{l=1}^{L}\sum_{m=1}^{M}{\frac{\left(p_{l,m}-\zeta \left(\pi \left(R_{m}\left(P_{l}-C_{m}\right)\right)\right)\right)}{\sigma_{im}^{A}}}^{2} \end{array}$$

(2) $$\begin{array}{c} +\sum_{z=1}^{Z}\sum_{k=1}^{K}{\frac{\left(R_{k}\left(C_{k}-C_{\mathrm{gnss},k}\right)-{\vec{\theta }}_{z}\right)}{\sigma_{gnss}}}^{2} \end{array}$$

(3) $$\begin{array}{c} +\sum_{n=1}^{N}{\frac{\left(P_{n}-P_{\mathrm{gcp},n}\right)}{\sigma_{gcp}}}^{2} \end{array}$$

(4) $$\begin{array}{c} +\sum_{n=1}^{N}\sum_{m=1}^{M}{\frac{\left(p_{n,m}-\zeta \left(\pi \left(R_{m}\left(P_{\mathrm{gcp},n}-C_{m}\right)\right)\right)\right)}{\sigma_{im}^{H}}}^{2} \end{array}$$

where:

*l* is the index of tie points;
*m* is the image index;
*z* is the index of image blocks assorted by lever-arm;
*k* is the index of images with GNSS measurements;
*n* is the index of GCPs;
*ζ* is the camera model;
*π* is the projection function;
*p_l,m_* is the 2D position of tie point *l* in image *m*;
(*R*m**, *C_m_*) is the pose of image m;
*P_l_* is the 3D position of tie point *l*;
*C_k_* is the camera projection center of image *k*;
*C*_gnss,*k*_ is the phase center of GNSS antenna of image *k*;
*R_k_* is the world to camera rotation;
${\vec{\theta }}_{z}$ is the lever-arm of image block *z*;
*P_n_* is the pseudo-intersection position of GCP *n*;
*P*_gcp,*n*_ is the ground measurement of GCP *n*;
*p_n,m_* is the image measurement of GCP *n* in image *m*;
$\sigma_{\mathrm{im}}^{A}$ is the weight of tie points in images;
$\sigma_{\mathrm{gnss}}$ is the weight of GNSS measurements;
$\sigma_{\mathrm{gcp}}$ is the weight of GCPs; and
$\sigma_{\mathrm{im}}^{H}$ is the weight of image measurements of GCPs.

The global cost function is composed of four parts: (1) the cost of tie points; (2) the cost of GNSS measurements; (3) the cost of GCPs; and (4) the cost of image measurements of GCPs. Three strategies were implied here to determine the observation weight. The first strategy weights observations by their true standard deviation known a priori (similar to Gauss–Markov model). The second strategy controlled observation weights of each category, which avoids overweighting one single category only due to its abundance (e.g., tie point observations are abundant compared to GCP observations). The third strategy handled robustness; a higher weight was given to observations having small residuals during BBA procedure. The minimization problem was solved with Levenberg–Marquardt (L-M) method. The L-M is in essence the Gauss–Newton method enriched with a damping factor to handle rank-deficient Jacobian matrices [27].

Typically, a single lever-arm was estimated per image block. Here, to deal with an image block of multiple flight configurations (i.e., nadir and oblique camera-looking flights), the cost function was extended to take into account multiple lever-arms in one image block.

## 4. Data Acquisition

### 4.1. Acquisition Field

On 4 October 2017, several flights were conducted in Culoz, France. The surveying object of interest is a north–south orientated dike of about 1.2km long with a turn on the north end. The scene has a corridor configuration with little height difference. Thirty-one ground points were regularly placed along the corridor and surveyed to be used either as ground control points (GCP) or check points (CP).

### 4.2. Flight Design

According to the flight authorization issued by the DGAC (Direction Générale de l’Aviation Civil), the distance between the telepilot and the employed UAV could not surpass 600 m during the acquisition. Therefore, the acquisition field was divided into two segments of 600 m and surveyed separately. The first segment of 600 m consists of the south part of the dike, while the second segment consists of the rest of the dike including the turn at the north end.

The first segment was surveyed with the routine acquisition configuration, a nadir flight of 3 strips at 50 m (denoted as *s1-n50*). This configuration is simple and economic, whereas not preferable when high photogrammetric accuracy is demanded. It introduces correlations among the camera focal length, the height of camera and the lever-arm. Consequently, parameters could not be accurately estimated. The second segment consists of three flights, a nadir flight of 3 strips at 50 m (denoted as *s2-n50*), an oblique flight of 3 strips at 50 m (denoted as *s2-o50*) and a nadir flight of 2 strips with the first strip at 70 m and the second strip at 30 m (denoted as *s2-n3070*). When mixing oblique and nadir images, images acquired from different flight heights, the above-mentioned correlations could be significantly mitigated and these parameters were better estimated. This configuration of multiple acquisition angles and multiple flight heights is desirable though costly. The objective of the study was to investigate, given a good camera model and a correct lever-arm, the achievable accuracy of a simple while not preferable network configuration. The estimated camera model and lever-arm of the image block of segment 2 (*s2-n50* + *s2-o50* + *s2-n3070*) was considered of high accuracy and was used for sensor pre-calibration of the image block of Segment 1 (*s1-n50*).

**Figure 3:**
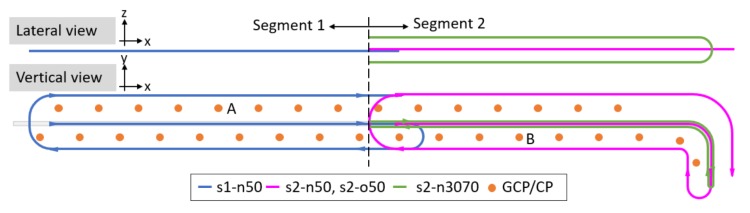
An illustration of the conducted flights.

Figure 3 depicts the conducted flights and the information of flights are given in Table 1.

## 5. Data Processing

### 5.1. Topographic Data Processing

The measurements of ground points were carried out with a Leica total station. Seven stations in total were employed in “free” station mode and the measurements were performed over two days. A Leica Viva GNSS receiver was installed on the south end of the dike to give absolute georeferencing information. Its position was precisely determined by processing a ∼26 h static observation session over two days with the service provided by the IGN. The compensation of the topographic measurements was carried out with Comp3D, a geodesic micro-compensation software developed at IGN.

### 5.2. GNSS Data Processing

The GNSS raw data were post-processed by RTKLib open source software in the carrier-phase differential mode with respect to the Leica Viva GNSS receiver mentioned in Section 5.1 as base station. Table 2 presents the configuration of main parameters used in RTKLib for the GNSS trajectory processing. The ratio of epochs with a fixed solution to total epochs of the GNSS trajectory solution is 99.17% and the mean standard deviation along *x*, *y* and *z* axes amounts to 8.4, 5.5 and 9.6 mm, respectively.

### 5.3. Synchronization of GNSS and Camera Modules

**Figure 4:**
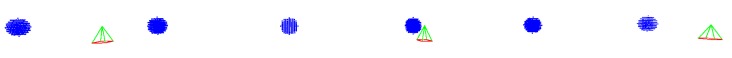
An illustration of the desynchronization between GNSS (blue circles) and camera (pyramid) modules.

With a GPS sampling rate of 1 Hz, a position is calculated every second. Nevertheless, the frequency of image acquisition is 0.4 Hz, which introduces a desynchronization between GNSS and camera system. To determine the position of the camera at exposure, a parabolic interpolation was carried out taking into account the GNSS-derived position accuracy and velocity. Figure 4 depicts the desynchronization between the two systems; circles in blue represent GNSS-derived positions and pyramids represent camera frames.

### 5.4. Photogrammetric Data Processing

The photogrammetric data processing is performed with MicMac, a free open-source photogrammetric software developed at IGN and ENSG (Ecole Nationale des Sciences Géographiques) since 2003 [25].

**Figure 5:**
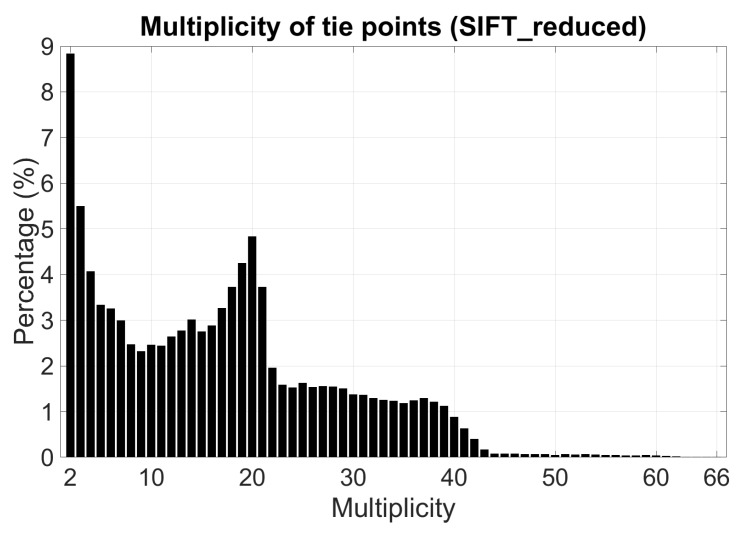
Multiplicity histogram of SIFT-reduced tie points.

**Figure 6:**
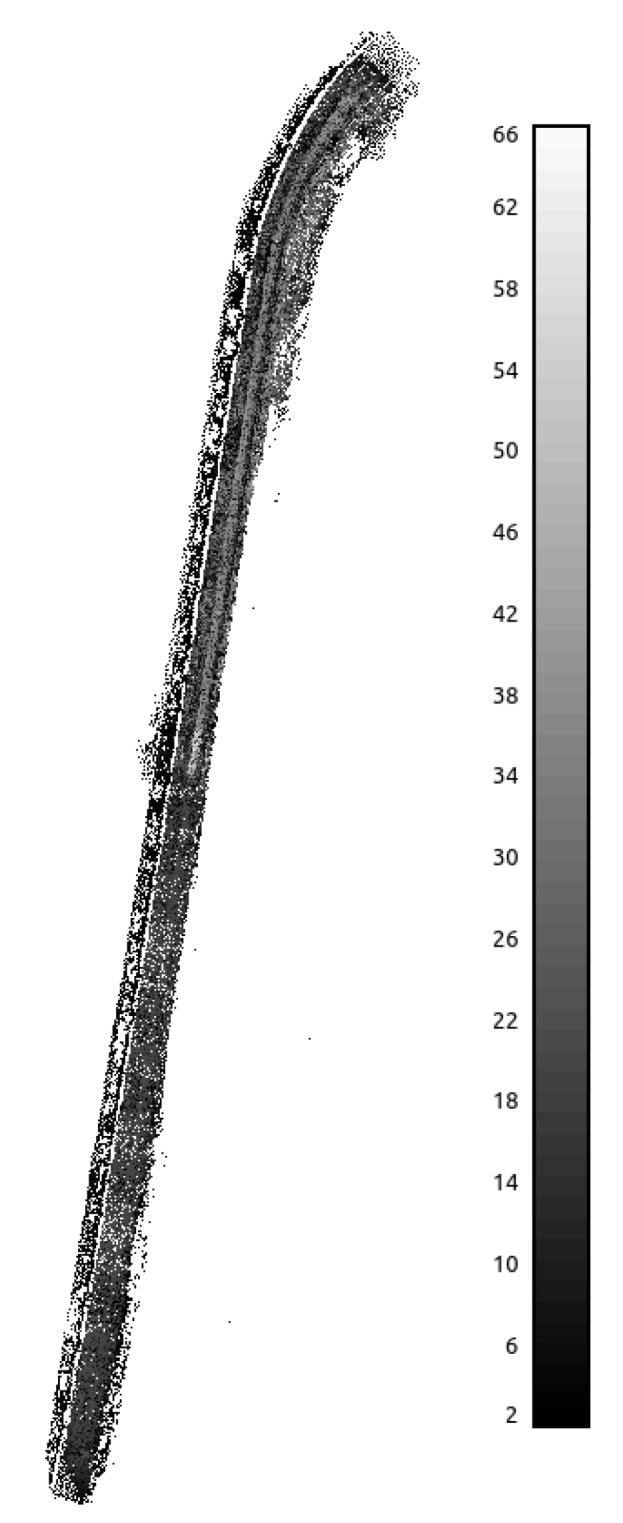
Distribution of SIFT-reduced tie points with multiplicity represented by grey scale.

To reduce memory requirements and processing time, a tie point reduction is then performed on SIFT-extracted tie points while maintaining tie point multiplicity with a stand-alone tool in MicMac [28]. Figure 5 and Figure 6 depict the distribution and multiplicity of SIFT-reduced tie points.

**Figure 7:**
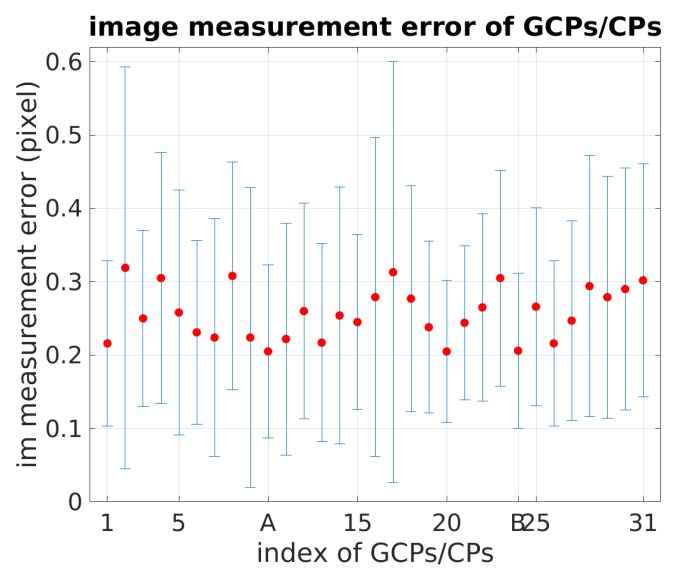
Image measurement error of GCPs/CPs.

Image measurements of GCPs/CPs are performed manually. Figure 7 depicts the image measurement error on GCPs/CPs ordered according to their position along the south–north direction (point A: 10th, point B: 24th).

## 6. Results

**Figure 8:**
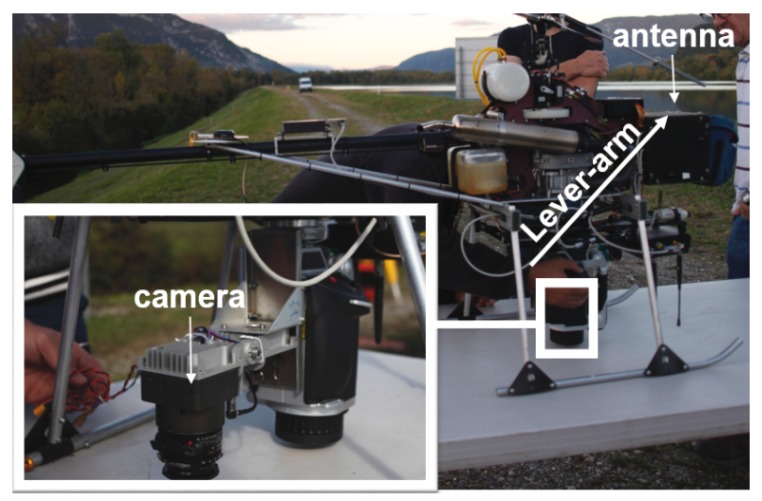
An illustration of the relative position between the camera and the GNSS receiver antenna.

The photogrammetric data processing results are reported in Table 3. Flights of Segment 2 (s2-n50, s2-o50, and s2-n3070) were used for camera and lever-arm calibration. The camera model and lever-arm were considered unknown and were estimated during the BBA procedure. The accuracy was evaluated with CPs and the root-mean-square (RMS) of residuals on CPs was used as the accuracy criteria (the GCPs included in the BBA procedure were not used as CPs for accuracy evaluation). The camera model and lever-arm that gave better accuracy were then used as a priori information for Flight s1-n50. For each image block, the photogrammetric accuracy and estimation results were given in two cases: without GCP and with one GCP used in the BBA procedure. The internal photogrammetric accuracy was measured by σ, the standard deviation of all image measurement residuals for the entire block. The lower σ, the less tension within the BBA solution. Typically, a σ in the range of 4–7 μm (0.6–1 pixel) is an indication of a high quality internal block adjustment. The external photogrammetric accuracy is measured by the RMS calculated on check point 3D coordinates (GCP used during BBA is excluded). Estimated lever-arms are given in the camera frame (the original point is the camera optical center). Figure 8 depicts the relative position between the GNSS receiver antenna and the camera body, which corresponds to the lever-arm vector.

Given the little elevation difference along the dike, a strong correlation exists between the camera focal length, the height of the camera and the *z* component of the lever-arm. The correlation coefficients of these parameters are given for each configuration.

In the following, the results are discussed in more details focusing on the influence of the adopted acquisition geometry on the final accuracy in object space.

### 6.1. Influence of Oblique Images

Two datasets were processed for camera and lever-arm calibration. The first dataset combines nadir and oblique images of Flights s2-n50 and s2-o50. The inclusion of oblique images eases the correlation between the focal length and the lever-arm as well as between the focal length and the camera height ($\delta_{LA_{z},f}$ = 0.18 and $\delta_{C_{z},f}$ = 0.18). However, when no GCP is included in BBA procedure, the *z* component of lever-arm remains highly correlated with the camera height ($\delta_{C_{z},LA_{z}}$ = 0.99). By including one GCP (Point B, as shown in Figure 3), the *z* component of lever-arm is better decorrelated from the height of camera (correlation coefficient $\delta_{C_{z},LA_{z}}$ = 0.80). With the added information, more constraints are applied within the BBA procedure and the correlations between the focal length and other two parameters increased ($\delta_{LA_{z},f}$ = 0.84, $\delta_{C_{z},f}$ = 0.68). The internal photogrammetric accuracy is also slightly improved (σ is decreased by 0.3 μm). Moreover, the RMS of residuals on CPs decreases considerably, especially along *z*-axis. This gain is mainly due to the proper estimation of the *z* component of lever-arm. We can see that the RMS of residuals on CPs decreases by 7.6 cm on vertical direction while the estimated lever-arm vector has an increase of 7 and 6.1 cm on *z* axis for nadir and oblique flight, respectively.

### 6.2. Influence of Multiple Flight Heights

In the second dataset, nadir images of different flight heights (s2-n3070) are added to the first dataset (s2-n50, s2-o50). Without any GCP, the addition of images of different flight heights eases the correlation of the focal length with other two parameters ($\delta_{LA_{z},f}$ = 0.07 and $\delta_{C_{z},f}$ = 0.07) while the lever-arm on *z*-axis is still strongly correlated to camera height ($\delta_{C_{z},LA_{z}}$ = 0.99). The RMS of 3D point residuals is improved by 2.4 cm and the lever-arm estimation of nadir image is diminished by 2 cm. With one GCP being included, the addition of images of different flight heights largely reduces the correlations among these three parameters ($\delta_{LA_{z},f}$ decreases from 0.84 to 0.55, $\delta_{C_{z},LA_{z}}$ decreases from 0.80 to 0.62, and $\delta_{C_{z},f}$ decreases from 0.68 to 0.33). The internal and the external photogrammetric accuracies are further improved and the RMS of 3D point residuals is decreased to 1.0 cm. For camera model and lever-arm calibration, all GCPs of Segment 2 are included for computing an optimal BBA solution. Parameter estimates issued from this BBA procedure are then used as a priori information for the Flight s1-n50.

### 6.3. Basic Flight Configuration

For a simple, economic while not preferable flight configuration such as s1-n50, with neither a priori information on the camera model and the lever-arm, nor any GCP being included in the BBA procedure, an important residual is observed on the *z* component. The camera height and the lever-arm are strongly correlated ($\delta_{C_{z},LA_{z}}$ = 0.99). The inclusion of one GCP (Point A, as shown in Figure 3) improves to a large extent the photogrammetric accuracy, especially on the *z* axis. However, due to the poor geometry of the acquisition network, the correlation between the lever-arm and the height of camera remains high. Though residuals are small on *z* axis, the *z* component of the lever-arm is far from being correct. The accuracy is less satisfying than when oblique images are also taken into account.

Given a good calibration of the camera model and the lever-arm estimated with the image block of Segment 2 (s2-n50 + s2-o50 + s2-n3070) with all GCPs, the photogrammetric accuracy is further improved. Without any GCPs, the RMS of 3D point residuals on CPs is equal to 3.9 cm. It is worth noting that when giving well calibrated camera and lever-arm, the results obtained with a nadir flight of 3 strips (s1-n50 + 0 GCP) outperform that obtained with the second dataset (s2-n50 + s2-o50 + s2-n3070 + 0 GCP). The inclusion of one GCP does not improve the external photogrammetric accuracy on *z*, which can be explained by the fact that the camera model and lever-arm are already well-calibrated and fixed during the BBA procedure. However, even with well-calibrated parameters, the accuracy of image block s1-n50 does not exceed the one of the image block s2-n50 + s2-o50 + s2-n3070 when employing one GCP. It is possibly due to the better quality (better multiplicity and distribution) of tie points extracting from the latter image block.

## 7. Discussion

Four flights were conducted on two segments of a 1.2 km long dike and about 1100 images were acquired. In this study, unlike what most commercially available photogrammetric softwares do, we performed an estimation of multiple lever-arms within the BBA procedure. It liberates us from the physical constraint between the camera and the GNSS antenna and makes it possible to merge image blocks of different configurations. For camera calibration strategy, instead of performing laboratory calibration in a close range configuration, we proposed an in-flight calibration which describes the true acquisition condition and gives more accurate camera calibration. Despite the poor geometry of the dike, our approach obtained a centimetric accuracy with one GCP and an accuracy of 3.9 cm with 0 GCP when in-flight calibration of camera and lever-arm is provided. The results outperform that of the majority of literature works.

Based on the experiments presented in the article, we suggest that the best acquisition geometry for corridor mapping is the inclusion of nadir and oblique images of different flight heights. The mapping accuracy can reach 1 cm when good GNSS data and at least one GCP are given. For the acquisitions with lower accuracy demands and limited budget, one nadir flight is sufficient for achieving an accuracy of 4 cm with a well-calibrated camera and lever-arm. Under the condition where well-calibrated camera and lever-arm are unavailable, the combination of one nadir and one oblique flight can reach an accuracy of 8–10 cm without any GCPs.

## Figures and Tables

**Table 1:** Details on the conducted flights.

Flight		*s1-n50*	*s2-n50*	*s2-n3070*	*s2-o50*
Nb of images		395	315	200	323
Height (m)		50	50	30, 70	50
Orientation		nadir	nadir	nadir	oblique
Nb of strips		3	3	2	3
Overlap (%)	forward	80			
	side	70			
GCP accuracy (mm)	horizontal	1.3			
	vertical	1			
Camera focal length (mm)		35			
GSD (mm)		10	10	6, 14	10

**Table 2:** RTKLib parameterization of GNSS trajectory processing.

Positioning Mode	Kinematic	Troposphere Correction	Saastamoinen
Frequencies	L1	Satellite Ephemeris	Broadcast
Filter type	Combined	Navigation System	GPS
Elevation Mask	15°	Integer Ambiguity Resolution	Fix and Hold
Ionosphere Correction	Broadcast	Min Ratio to Fix Ambiguity	3.0

**Table 3:** Results of photogrammetric data processing.

	GCP	Parameter Liberation	σ [μm]	RMS [cm]			Lever-Arm [cm]				Correlation		
				*xy*	*z*	3D		*xy*	*z*	3D	$\delta_{LA_{z},f}$	$\delta_{C_{z},LA_{z}}$	$\delta_{C_{z},f}$
**Calibration**													
s2-n50 + s2-o50	0	camera model, lever-arm	5.2	1.9	8.3	8.5	na:	51.4	34.8	62.1	0.18	0.99 ± 0.00	0.18 ± 0.00
							ob:	63.2	12.7	64.5			
	1 (pt B)	camera model, lever-arm	4.9	0.9	0.6	1.1	na:	51.0	41.8	65.9	0.84	0.80 ± 0.06	0.68 ± 0.05
							ob:	66.2	18.8	68.8			
s2-n50 + s2-o50 + s2-n3070	0	camera model, lever-arm	5.0	1.9	5.9	6.2	na:	51.9	36.8	63.7	0.07	0.99 ± 0.00	0.07 ± 0.00
							ob:	64.8	14.6	66.4			
	1 (pt B)	camera model, lever-arm	4.7	0.7	0.8	1.0	na:	51.7	42.3	66.8	0.55	0.62 ± 0.09	0.33 ± 0.06
							ob:	66.5	19.4	69.3			
	14 (all)	camera model, lever-arm	4.7	/	/	/	na:	51.7	42.8	67.2	0.85	0.44 ± 0.09	0.39 ± 0.08
							ob:	66.6	19.9	69.5			
**Acquisition**													
s1-n50	0	camera model, lever-arm	4.8	3.3	15.0	15.3	na:	53.8	47.7	71.9	0.28	0.99 ± 0.00	0.28 ± 0.00
	1 (pt A)	camera model, lever-arm	4.8	3.3	0.9	3.4	na:	54.1	33.0	63.4	0.92	0.98 ± 0.02	0.91 ± 0.01
	0	N/A, given	5.2	3.3	2.1	3.9	na:	51.7	42.8	67.2	/	/	/
	1 (pt A)	N/A, given	5.1	3.4	2.1	4.0	na:	51.7	42.8	67.2	/	/	/

σ represents the sigma naught in bundle adjustment; $\delta_{LA_{z},f}$ represents the correlation coefficient between the nadir flight lever-arm on *z*-axis and the focal length; $\delta_{C_{z},LA_{z}}$ represents the correlation coefficient between the camera height and the nadir flight lever-arm on *z*-axis; $\delta_{C_{z},f}$ represents the correlation coefficient between the camera height and the focal length; and N/A means the camera model and the lever-arm are not estimated but given as a priori informations

## Abbreviations

The following abbreviations are used in this manuscript:

GNSS	Global navigation satellite system
BBA	Bundle block adjustment
ISO	Integrated sensor orientation
UAV	Unmanned aerial vehicles
GCP	Ground control points
INS	Inertial navigation system
IMU	Inertial measurement unit
CP	Check point
Loemi	Laboratoire d’opto-éléctrique de métrologie et d’instrumentation
IGN	Institut national de l’information géographique et forestière
SIFT	Scale Invariant Feature Transform
DGAC	Direction générale de l’aviation civil
ENSG	Ecole Nationale des Sciences Géographiques
RMS	Root mean square
